# Effect of conventional physiotherapy versus strain counter for trapezitis patients

**DOI:** 10.6026/9732063002001410

**Published:** 2024-10-31

**Authors:** Alok Desai, Anandh S., Dhirajkumar Mane

**Affiliations:** 1 Krishna College of Physiotherapy, Krishna Vishwa Vidyapeeth (DU), Karad, Malkapur, Karad, Maharashtra, India; 2 Department of Community Health, Krishna College of Physiotherapy, Krishna Vishwa Vidyapeeth (DU), Karad, Malkapur, Karad, Maharashtra, India; 3 Statistician, Directorate of Research, Krishna Vishwa Vidyapeeth (DU), Karad, Malkapur, Karad, Maharashtra, India

**Keywords:** Physiotherapy, strain-counterstain (SCS), tirapazamine, pain, neck disability

## Abstract

On average 38.7% of the adult population experiences neck pain due to trapezitis. Therefore, it is of interest to determine the efficacy of strain-counterstain (SCS) technique in a treatment of trapezitis. 40 participants were randomly allocated to two groups, where group 1 received conventional physiotherapy treatment, while group 2 received the experimental SCS treatment program with 20 patients each. We found that, group 2 showed significant improvements in range of motion, pain and neck disability outcome scores. Thus, that SCS can be used as a low-cost and effective treatment for trapezitis.

## Background:

A study has shown that trapezitis is characterized by acute or persistent pain in the neck and shoulders. Furthermore, it is estimated that approximately 38.7% of the adult population suffers from neck pain attributable to trapezitis [1]. A study has indicated that monotonous jobs characterized by highly repetitive tasks, forceful exertions, elevated levels of static contractions, prolonged static loads, constrained work postures, or a combination of these factors may contribute to the development of neck and shoulder disorders, including trapezitis, within the working population [2]. Of the 20% of persons who suffer from severe chronic pain in the shoulder and neck regions, 10% to 20% have persistent TP. Among women, especially in low-income groups, the PS variant of trapezitis has a higher prevalence. The much greater prevalence in women indicates that gender plays a significant role in the development of neck diseases. In comparison to males, women are more prone to have chronic neck pain and are more often impacted by it [2]. Studies have also shown that, the content of their jobs might explain this difference [3, 4]. A study has shown some evidence for the short-term relief of myofascial trigger points using Transcutaneous Electrical Nerve Stimulation (TENS) [5]. Ultrasound therapy may be as effective as a placebo or just slightly more effective than conventional treatments for myofascial trigger points, according to a study that has shown conflicting evidence about its effectiveness [6]. The use of ultrasound as a therapeutic modulator has been shown in a study, although it is not recommended for people of any age [6]. Addition to this, studies have shown that, stretching of the upper trapezitis muscle appears to have an instant improvement in pain [5, 6]. Another study showed that it is utilized worldwide as a result of its effectiveness, simplicity, low cost, and ease of shipment. It also reduces edema, nerve conduction rates, cellular metabolism, and local blood flow [7]. Another study found that the Strain Counter Strain (SCS) is an osteopathic technique that employs indirect manipulation to alleviate pain and restore muscle, bone, and joint function [8]. SCS is utilized to control sensitive points in such a way that the pain is reduced by at least 70% in order to determine the position of ease. It is suggested that the minimum duration required to sustain a position of ease is 90 seconds [9]. Studies have also shown that proprioceptive and nociceptive processes work together to shorten or fold over aberrant tissues in positional release [9, 10]. Therefore, it is of interest to evaluate the efficiency of SOS technique for treating trapezitis patients.

## Methods:

The current study was conducted at Krishna College of Physiotherapy. 40 subjects were further divided into 20 subjects each. The assessment was taken before the treatment using the outcome measures. The patients were assessed for pain, cervical ROM and neck disability. Visual Analogue Scale (VAS) was used to assess pain at rest and on activity. It is a widely accepted and reliable scale for pain assessment indicating no or minimum pain at 0 and maximum pain at 10. Similarly, cervical lateral flexion was measured using a goniometer. Neck disability was assessed using the Neck Disability Index (NDI). Group 1 received conventional physiotherapy treatment 3 times a week for 4 weeks. Ultrasonography was set at 0.8 W/cm^2^ and was applied for 15 minutes. Cryotherapy was administered using the direct application of an ice pack for 15 minutes. Frequency was set to 100 Hz and duty cycles of 250s. Group 2 had SCS technique treatment three times per week for four weeks. To lessen the reported transient receptor potential (Trp) pain, the SCS technique was provided to the patients in supine lying while the practitioner positioned the ipsilateral arm in flexion, abduction, and external rotation at weeks 1 and 2. Once the position of ease has been found, pressure is given to the Trp for 20-30 seconds before being repeated three times. Cryotherapy was administered using the direct application of an ice pack for 15 minutes. At week 3 & 4, the SCS technique was used with the patients in supine lying while the practitioner positioned the ipsilateral arm in flexion, abduction and external rotation to reduce the reported Trp pain. Once the position of ease has been established, pressure is given to the Trp for 30-40 seconds and then repeated five times. Cryotherapy was administered using the direct application of an ice pack for 15 minutes.

## Statistical analysis:

Using IBM SPSS Version 23, pre- and post-intervention scores for all outcomes were compared using the unpaired 't' test, with a significance level set at 95%.

## Results:

Comparisons were made between the subjects in both groups who completed the trial using an unpaired 't' test (n=20 versus n=20). Table 1 shows that, both at rest and on activity showed statistically significant difference as the p value was >0.001 respectively. Table 2 shows that, both the variables showed statistically significant difference as the p value was >0.001 respectively. Table 3 shows that, both the variables showed statistically non-significant difference as the p value was 0.055 for group 1 while 0.051 for group 2 respectively. Table 4 shows that, both the variables showed statistically non-significant difference as the p value was 0.0204 for group 1 while significant difference >0.001 for group 2 respectively.

## Discussion:

study have shown that, around 38% of the adult population suffers from neck pain due to trapezitis, thus it is essential to find an effective and cost-efficient treatment for this burden on society [1]. It was found in our study that, there was a statistically significant difference in the reduction of pain, CROM and NDI scores between Group 1 and 2. Group 2 showed significant improvement across all outcomes. Moreover, treatment protocols for managing trapezitis can be developed inculcating SCS as the primary intervention. This would not only, help to reduce the treatment time but also, to reduce the cost of treatment. As this treatment does not entail using any electrical modalities, the chances of electrical mishaps are greatly reduced. The limitation of the study includes small sample size, short duration of research and patients that were recruited were limited to only 1 geographical location. It has been shown that both strain-counterstrain and ischemic compression can help people with neck pain caused by trigger points in the upper trapezius muscle reduce their pain and disability index scores and improve their cervical range of motion in lateral flexion [11]. Both PRT and TENS are helpful in lowering pain in patients who have trapezitis. When it comes to relieving the pain associated with trapezitis, however, TENS results in less effective pain relief than PRT [12]. In patients with upper trapezius trigger points, a combination of conventional physical therapy and strain counterstrain is somewhat more effective than conventional physical therapy alone in terms of reducing pain and functional impairment and increasing cervical range of motion. The results of this research contribute to an increasing body of information supporting the use of strain counterstrain as a component of the treatment of myofascial trigger points [13].

## Conclusion:

Data shows that group 2 showed statistically significant improvement in individuals diagnosed with trapezitis. Thus, SCS can be used as a low-cost at effective treatment for trapezitis. The improvements acquired during this study were not traced for longer duration; hence we cannot mention the enduring effect of the treatment protocol.

## Figures and Tables

**Table 1:** Comparison of group 1

	**Pre-intervention Mean± SD**	**Post-intervention Mean± SD**	**p-value**
At rest	4.1± 0.7	2.7± 0.6	> 0.001
On activity	6.1± 0.8	3.5± 0.5	> 0.001

**Table 2:** Comparison group 2

	**Pre-intervention Mean± SD**	**Post-intervention Mean± SD**	**p-value**
At rest	3.6± 0.5	1.9± 0.4	> 0.001
On activity	6.2± 0.7	2.8± 0.5	> 0.001

**Table 3:** Comparison b/w group 1 & 2

	**Pre-intervention Mean± SD**	**Post-intervention Mean± SD**	**p-value**
Group 1	27.8± 5.8	36.6± 6.0	0.055
Group 2	26.7± 5.0	37.8± 5.5	0.051

**Table 4:** Comparison of group 1 & 2

	**Pre-intervention Mean± SD**	**Post-intervention Mean± SD**	**p-value**
Group 1	12.4± 3.9	7.2± 3.1	0.0204
Group 2	14.1± 3.4	6.1± 3.2	> 0.001

## References

[1] Kumar GY (2015). International journal of physical education, sports and Health..

[2] Meseguer AA (2006). Clinical chiropractic..

[3] Adam Perreault Med AT (2009). Athletic Training & Sports Health Care..

[4] Chorsiya V (2013). International Journal of Scientific Research..

[5] Alghadir AH (2020). Bio Med research international..

[6] Jung JH (2011). Physical Therapy Korea..

[7] Segura-Ortí E (2016). Acupuncture in Medicine..

[8] Patel VD (2018). Hong Kong Physiotherapy Journal..

[9] Ellythy MA (2012). Bull Fac Phys Ther..

[10] Metgud SC (2020). Indian Journal of Physical Therapy and Research..

[11] Gohil D (2020). Archives of Medicine and Health Sciences..

[12] Ramteke J (2018). Int J of Allied Med Sci and Clin Res..

[13] Javaid HM (2016). Annals of King Edward Medical University..

